# *In vivo* administration of G9a inhibitor A366 decreases osteogenic potential of bone marrow-derived mesenchymal stem cells

**DOI:** 10.17179/excli2019-1234

**Published:** 2019-06-03

**Authors:** Hedyeh Khanban, Esmail Fattahi, Mahmood Talkhabi

**Affiliations:** 1 Department of Biology, Ayatollah Amoli Branch, Islamic Azad University, Amol, Iran; 2 Department of Animal Sciences and Biotechnology, Faculty of Life Sciences and Biotechnology, Shahid Beheshti University, Tehran, Iran

**Keywords:** bone marrow-derived mesenchymal stem cells, histone methyltransferase G9a, adipogenesis, osteogenesis, A366

## Abstract

Epigenetic mechanisms such as histone methylation are considered as one of the most important mediators that control stem cell behaviors such as proliferation, senescence and differentiation. G9a, a histone methyltransferase, has recently generated intense attention as potential target for controlling many diseases such as cancers. The aim of the present study was to evaluate the effect of *in vivo* administration of A366, a G9a inhibitor, on proliferative and differentiation potential of bone marrow-derived mesenchymal stem cells (BM-MSCs). We inhibited G9a using intraperitoneally administration of A366, and we evaluated BM-MSC proliferation and differentiation behaviors *in vitro*. Colony formation assay of BM-MSCs at primary culture showed that *in vivo* administration of A366 reduced the colony forming capacity of BM-MSCs. Moreover, PDT of BM-MSC isolated from A366-treated rats was higher than control, especially in the early passages. BM-MSC isolated from A366-treated rats showed higher adipogenic potential compared to the control at the early passages as determined by gene expression and Oil Red staining. Whereas, osteogenic potential of BM-MSC isolated from A366-treated rats was lower than control, especially at early passages. Our results suggest that the epigenetic modifier such as A366, which seems to be a therapeutic approach for controlling diseases such as cancer, might also influence the proliferation and differentiation capacity of MSCs both *in vitro* and *in vivo*. Moreover, epigenetic modifying chemicals seem to be a strategy to manipulate MSC expansion capacity and differentiation propensity, as well as to efficiently involvement of MSCs in tissue homeostasis, cell-based therapy and tissue engineering.

## Introduction

Mesenchymal stem cells (MSCs) also called mesenchymal stromal cells are a heterogeneous population of fibroblast-like cells of non-hematopoietic origin, which have been isolated from a variety of connective tissues such as bone marrow (Fitzsimmons et al., 2018[6]). MSCs easy isolation, simple culture, multiple isolation source, differentiation potential and secretory and immunomodulatory capacity of MSCs, make these cells as golden choice for cell-based therapy, tissue engineering, pharmaceutical industry and other potential applications (Liubaviciute et al., 2018[11]). 

Today it is well-known that epigenetic mechanisms control the stem cell behaviors (e.g. secretion, differentiation and proliferation) during homeostasis, aging, and disease (Beerman and Rossi 2015[3]; Ghasemzadeh et al., 2018[7]). Histone methylation is one of the epigenetic mechanisms that can strongly affect stem cell behaviors by activating or repressing gene expression. Histones methylations occur by several histone methyltransferases (HMTs) and methylations are dynamically removed by histone demethylases (HDMs) (Cho et al., 2015[5]). G9a, a HMT belonging to the Su(var)3-9 family, is responsible for histone H3 lysine 9 (H3K9) mono- and demethylation (H3K9me1/2) *in vivo* and *in vitro* (Casciello et al., 2015[4]). It is important to note that G9a is overexpressed in a number of cancers, including esophageal squamous cell carcinoma, hepatocellular carcinoma, aggressive lung cancer, brain cancer, multiple myeloma, and aggressive ovarian carcinoma (Casciello et al., 2015[4]). Since these epigenetic modifications are reversible, HMTs can be a potential target for controlling cancers. In addition to the G9a roles in cancer, it has an important role at normal development and stem cells differentiation. Although different functions have been reported for G9a during development, stem cell differentiation and cancers (Casciello et al., 2015[4]; Pappano et al., 2015[14]; Wang et al., 2013[17]), but it isn't understood the effects of* in vivo *G9a inhibition, as a potential strategy for cancer treatment, on proliferation properties and differentiation potential of bone marrow-derived mesenchymal stem cells (BM-MSCs) *in vitro* and *in vivo*.

Here, we injected intraperitoneally a dose of 25 mg/kg A366 to wistar rats (a single time). Then, the clonogenicity, population doubling time, adipogenic and osteogenic differentiation potential, and cell cycle were analyzed for the BM-MSC isolated from A366-treated and control rats. Our results showed that *in vivo* administration of A366 decreased proliferation capacity and increased adipogenic potential of BM-MSCs. While, osteogenesis was reduced following *in vivo* administration of A366.

## Materials and Methods

### In vivo chemical treatment and cell culture

To investigate the effects of *in vivo* administration of A366 on proliferation and differentiation potential of BM-MSCs, a single dose of 25 mg/kg of A366 (dissolved in DMSO and diluted with water) was injected intraperitoneally in rats (purchased from Royan Institute, Tehran, Iran). Control rats were given DMSO diluted with water. 2 days after IP injection, A366-treated rats and controls were sacrificed and the bone marrow cells were isolated as we previously described (Baghaban Eslaminejad et al., 2008[2]). Animal protocols were approved by the Institutional Animal Care and Use Committee at Shahid Beheshti University (SBU). BM-MSCs were cultured with MSC medium (Dulbecco's Modified Eagles Medium (Fisher Scientific) supplemented by 15 % Fetal Bovine Serum (Sigma Aldrich) and Penicillin-Streptomycin (Gibco) at 37 °C and 5 % CO_2_. The medium was changed with fresh medium every 2-3 days. In the present study, different passages of BM-MSCs were used (mentioned in each experiment). Population doubling time, cell-cycle profile and differentiation assay were analyzed for BM-MSCs isolated from treated and control rats.

### Confirming the identity of the cultured BM-MSCs 

Flow cytometry analysis (for surface markers CD90, CD73, and CD45) and differentiation assays (adipogenesis, osteogenesis and chondrogenesis) were used to confirm the identity of isolated BM-MSCs. To analyze the surface markers, BM-MSCs (at P3) were stained with FITC- or PE-conjugated anti-rat antibodies (BD, Bioscience), or with their isotypes, according the BD Bioscience protocol, then the cells were analyzed using a flow cytometer (FACSCalibur; BD Biosciences) and flowing software, version 2.5.1 (BD Biosciences). The MSC identity also was confirmed by differentiation of MSCs (at P3) to adipocyte, osteocyte and chondrocyte (Baghaban Eslaminejad et al., 2008[1]). Osteogenic, adipogenic and chondrogenic differentiation were determined using Alizarin Red, Oil Red, and toluidine blue staining, respectively.

### Cell population doubling time (PDT)

To determine the effect of *in vivo* administration of A366 on BM-MSC proliferation, 10^5^ BM-MSCs derived from A366-treated rats and controls, were seeded at each well of a 6-well plate. Cells were cultured for 4-6 days, and their media were changed every 48 hours, and PDT was calculated for the cells in each passage. PDT was examined using the formula: 


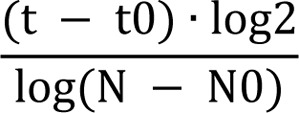


where t − t0 is culture time (h), N is the number of harvested cells and N0 is the number of cells in the initial.

### Cell cycle analysis

For analyzing cell cycle of BM-MSCs isolated from A366-treated and control rats, 10^5^ BM-MSCs (P1-P3) were seeded at each well of a 6-well plate. After 2 days, the cells were trypsinized and harvested, then cells were prepared for cell cycle analysis, according to a standard protocol (Moradi et al., 2017[13]). Flow cytometry was performed using a BD FACSCalibur (BD Biosciences) and the data were analyzed with BD FACSDiva (BD Biosciences).

### Osteogenic differentiation 

Osteogenic differentiation was induced using osteogenic differentiation medium (regular MSC medium supplemented with 50 mg/ml ascorbic 2-phosphate (Sigma-Aldrich), 10 nM dexamethasone (Sigma-Aldrich) and 10 mM ß-glycerol phosphate (Sigma-Aldrich) at P1-P3. Next, osteogenic differentiation was determined using Real-time PCR (see below) and Alizarin Red on day 21 of differentiation. Images were captured using a digital camera system (Olympus DP72) connected to an inverted optical microscope (Olympus CKX41-Olympus Optical CO). For quantitation the Alizarin Red staining, differentiated osteocytes were gently washed by PBS and fixed with 4 % paraformaldehyde in PBS (Sigma-Aldrich) for 15 min. Subsequently, the cells were washed with distilled water, exposed to Alizarin Red S (Sigma-Aldrich) for 20 min at room temperature, and washed again with distilled water for 5 min for 4 times. For quantification of Alizarin Red S staining, the dye was desorbed using 10 % (wt/vol) cetylpyridinium chloride (Sigma, C9002) for 1 hour and absorbance was determined at 540 nm using a Thermo Scientific™ Multiskan™ GO Microplate Spectrophotometer (Thermo Scientific™, USA). To analyze the ALP activity in osteocytes, Alkaline Phosphatase Assay Kit (abcam; ab83369) was used. ALP activity was determined based on the manufacturer's protocol and the results were measured at 405 nm (Thermo Scientific™, USA).

### Adipogenic differentiation

Adipogenic differentiation was induced using adipogenic differentiation medium (regular growth medium supplemented with 50 mg/ml ascorbic 2-phosphate (Sigma-Aldrich), 10 nM dexamethasone (Sigma-Aldrich) and 10 mM ß-glycerol phosphate (Sigma-Aldrich) at P1 and P2. After 3 weeks, the adipogenesis was assessed using Real-time PCR (see below) and Oil Red staining (Sigma Aldrich™). Images were captured using a digital camera system (Olympus DP72) connected to an inverted optical microscope (Olympus CKX41-Olympus Optical CO).

### Chondrogenic differentiation

To induce chondrogenesis, micro mass culture system was used (Baghaban Eslaminejad et al., 2008[1]). Briefly, 2.5×10^5^ BM-MSCs (at P3) was pelleted using centrifuge and cultured for three weeks in chondrogenic differentiation medium (DMEM supplemented with 10 % ITS+ Premix (Sigma-Aldrich), 10−7 M dexamethasone (Sigma-Aldrich), 1 μM ascorbate-2-phosphate (Sigma-Aldrich), 1 % sodium pyruvate (Sigma-Aldrich), 10 ng/ml BMP6 (Sigma-Aldrich) and 10 ng/ml transforming growth factor-beta 1 (TGF-β1, Sigma-Aldrich). At the end of differentiation, the chondrogenic aggregates were sectioned and stained using toluidine Blue, and viewed by light microscope. 

### RNA isolation and real-time PCR

To analyze the expression of osteogenic and adipogenic specific markers, MSCs were cultured for three weeks in osteocyte and adipocyte inducing medium, respectively. Then the cells were collected, RNAs were extracted, cDNA were synthesized and real-time PCR was performed using a standard procedure that we previously described (Talkhabi et al., 2015[16]). GAPDH was used as the internal control to normalize the expression of genes of interest. The results were presented as relative fold change compared to the control. The statistical significance was calculated by Student's t-test, and P values <0.05 were considered significant. The primer sequences for osteogenic and adipogenic genes and GAPDH are listed in Table 1(Tab. 1).

## Results

### Characteristics of BM-MSCs

**Figure 1:**
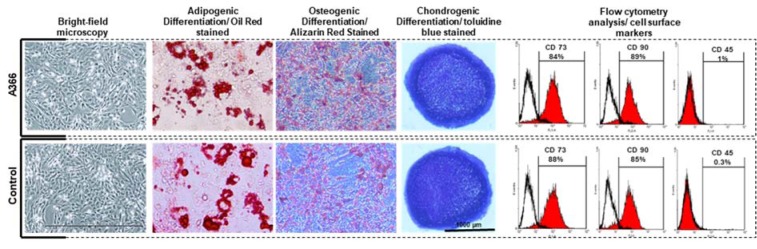
Characterization of BM-MSCs. BM-MSCs were isolated from A366-treated and control rats were assessed at P3. BM-MSCs isolated from both groups grew up as adherent cells with fibroblastic morphology. They had potential to differentiate to osteocytes, chondrocytes and adipocytes as determined by Alizarin Red, Toluidine Blue and Oil Red staining, respectively. They also were positive for CD73, CD90, and negative for the hematopoietic markers CD45.

The BM-MSCs isolated from A-366 treated rats and controls were expanded and cultured as plastic-adherent cells with a typical fibroblastic-like shape (Figure 1(Fig. 1)). The BM-MSCs isolated from A-366 treated rats and controls had capacity to differentiate into three important lineages including osteocytes, adipocytes and chondrocytes (Figure 1(Fig. 1)). Since BM-MSCs don't have any single specific marker determining their identity, so three known CD markers were analyzed for BM-MSCs. As expected, the majority of BM-MSCs were positive for CD90, CD73 (≃90 %), while most of the cells (≥ 98 %) were negative for CD45 (Figure 1(Fig. 1)). These three assessment confirmed that the isolated and investigated cells had MSC identity. 

### In vivo inhibition of G9a decreased clonogenicity and proliferation rate of BM-MSCs

**Figure 2:**
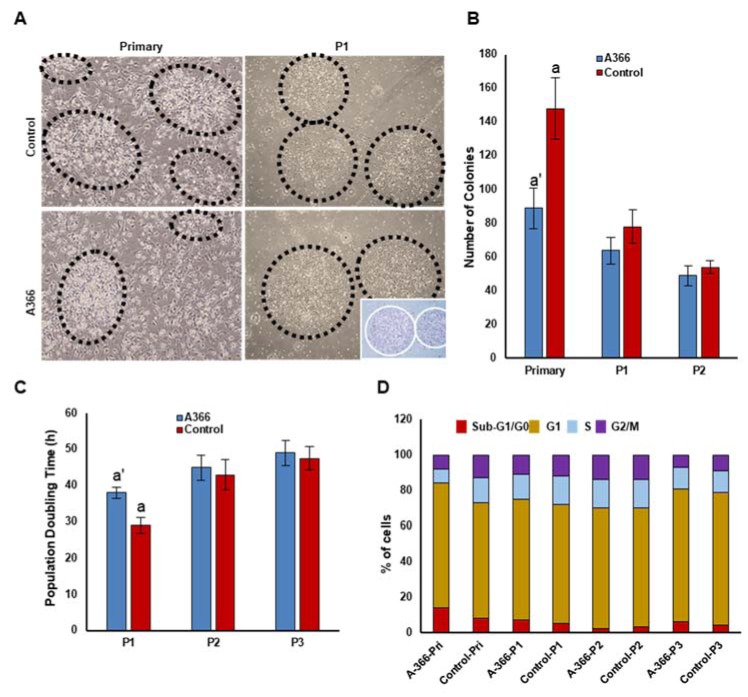
Effects of *in vivo* administration of A366 on clonogenicity and proliferative capacity of BM-MSCs. (A) Microscopic morphology of BM-MSCs at primary culture and P1. A366 decreased the clonogenicity of BM-MSCs at early passages; each black dotted circle is a colony of BM-MSC. Inset shows two colonies after Giemsa staining, (B) BM-MSC isolated from A366-treated rats formed significantly fewer colonies at primary culture *in vitro*, (C) BM-MSC isolated from A366-treated rats had significantly longer PDT than those isolated from control rats at the early passage, (D) cell cycle profile of BM-MSC; the proportion of cells at Sub-G1/G0 stage was larger in BM-MSC isolated from A366-treated rats than control, only at the primary culture. a': P < 0.05 vs a. Data are expressed as means ± SD.

To analyze the clonogenicity of BM-MSC isolated from A366 treated rats and controls, the number of MSC colonies formed at primary culture were counted after Giemsa staining (Figure 2A(Fig. 2)). We found that the number of colonies formed in the bone marrow cells isolated from A366-treated rats were significantly lower compared to the control (Figure 2B(Fig. 2)). To investigate whether inhibitory effect of A366 on BM-MSC clonogenicity can sustain during BM-MSC culture and expansion, we analyzed the colony forming capacity of BM-MSCs at the next passages. We found that at P1, the number of colonies formed by BM-MSC isolated from A366-treated rats was lower compared to the control (Figure 2B(Fig. 2)). Whereas, the difference between the number of colonies at P1 was not significant. There was also no significant difference between the number of colonies formed by BM-MSCs that were isolated from A366-treated rats and controls at P2 (Figure 2B(Fig. 2)) and P3 (n=2, data not shown). We also determined the PDT for BM-MSC isolated from A366-treated rats and controls at P1-P3. It was realized that at P1, the PDT of BM-MSC isolated from A366-treated rats was ~40 hours, which was approximately 13 hours longer than PDT of the control. Whereas, there was almost no difference in PDT of BM-MSCs at P2 and P3 (Figure 2C(Fig. 2)). Next, we analyzed the effect of *in vivo* administration of A366 on cell cycle profile. Therefore, cell cycle analysis of BM-MSC isolated from A366-treated rats and controls was calculated at primary culture and P1-P3. 

We found that in the primary culture, the proportion of cells at Sub G1/G0 stage was higher in BM-MSC isolated from A366-treated rats than control (Figure 2D(Fig. 2)). Whereas, the proportion of cells at G2 and S was lower in BM-MSC isolated from A366-treated rats than control (Figure 2D(Fig. 2)). Interestingly, the proportions of cells at Sub G1/G0, G1, S and G2/M were not significantly different between BM-MSC isolated from A366-treated rats and control at P1-P3.

### In vivo inhibition of G9a increased adipogenesis of BM-MSCs

**Figure 3:**
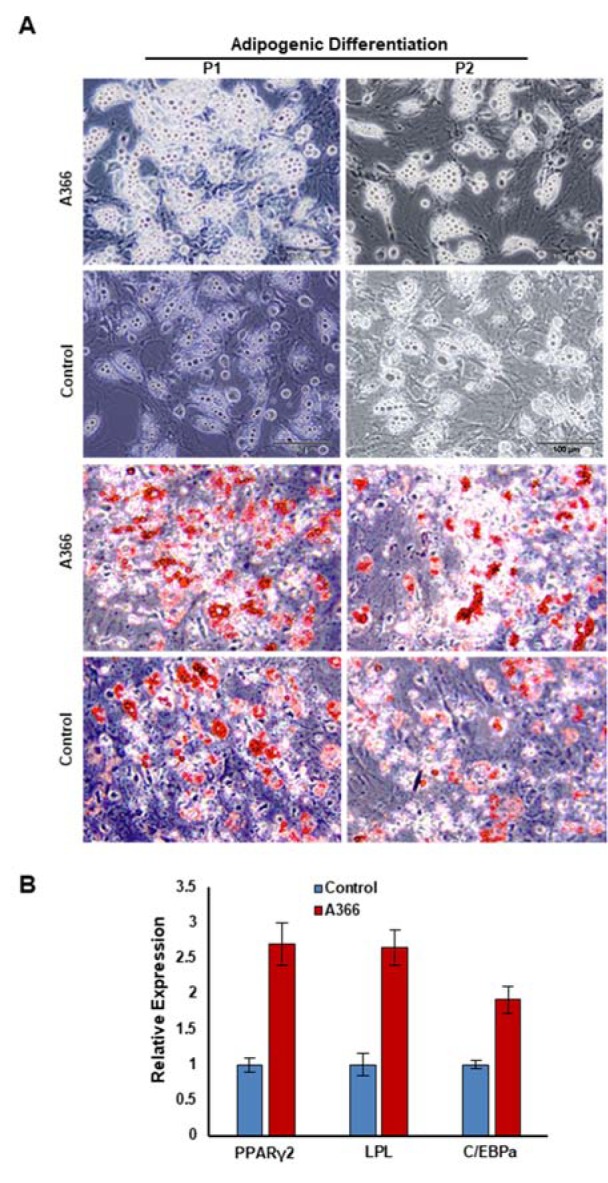
The effects of *in vivo* administration of A366 on adipogenic potential of BM-MSC*s*. (A) Most of BM-MSCs were differentiated to lipid droplet containing adipocytes in P1/BM-MSC isolated from A366 treated rats. BM-MSC isolated from A366-treated rats and control had same potential to generate adipocytes at P2 (Top). Oil red staining of the adipocytes differentiated from BM-MSC isolated from A366 treated and untreated rats (Bottom), (B) Gene expression analysis of BM-MSC-derived adipocytes. PPAR-γ2: peroxisome proliferator activated receptor gamma, LPL: lipoprotein lipase, C/EBPα: CCAAT Enhancer Binding Protein Alpha

Microscopic analysis showed that BM-MSC isolated from A366-treated rats had a higher potential to differentiate into lipid droplet containing adipocytes at the first passage (Figure 3A, top(Fig. 3)). However, there was approximately no difference between adipogenic differentiation of BM-MSC isolated from A366-treated rats and controls at P2 (Figure 3A, top(Fig. 3)) and P3 (data not shown). Consistent with microscopic observations, Oil Red staining of adipocyte differentiated from BM-MSCs at P1 revealed that *in vivo* administration of A366 increased the adipogenic potential of BM-MSCs *in vitro* (Figure 3A, bottom(Fig. 3)). We next analyzed the expression of adipocyte specific genes including peroxisome proliferator activated receptor gamma (PPAR-γ2), lipoprotein lipase (LPL) and CCAAT Enhancer Binding Protein Alpha (C/EBPα) in adipocytes differentiated from BM-MSC. We found that the expression of adipocyte specific genes was significantly higher in adipocytes differentiated from BM-MSC isolated from A366-treated rats than controls (Figure 3B(Fig. 3)).

### In vivo inhibition of G9a decreased osteogenesis of BM-MSCs

**Figure 4:**
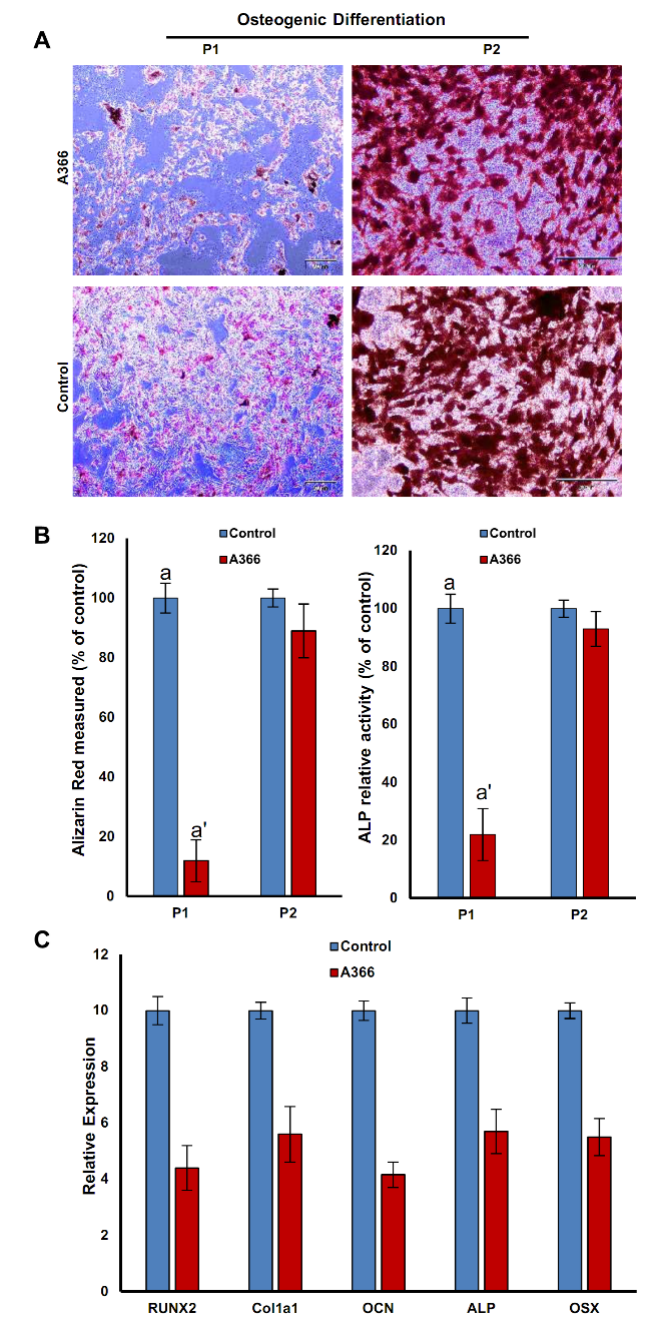
The effects of *in vivo *administration of A366 on osteogenic potential of BM-MSCs. (A) Alizarin red staining of BM-MSC-derived osteocytes. The osteogenic potential of the BM-MSC isolated from A366-treated rats clearly is lower than control at P1, whereas it is almost similar at P2; (B, left) Absorbance-based quantification of Alizarin Red staining showed that *in vivo* administration of A366 reduced the amount of extracellular matrix deposition by osteocytes differentiated from P1/BM-MSCs; (B, right) ALP activity was significantly lower in osteocytes differentiated from P1/BM-MSC that were isolated from A366-treated rats; (C) the gene expression analysis of osteocytes differentiated from BM-MSCs. a': P < 0.05 vs a. Data are expressed as means ± SD. Runx2: Runt-related transcription factor 2, OCN: Osteocalcin, ALP: alkaline phosphatase, OSX: Osterix and Col1a1: Collagen Type I Alpha 1 Chain

Osteogenesis assessment using Alizarin Red staining showed that the osteogenic differentiation in P1/BM-MSCs isolated from A366-treated rats severely decreased compared to the osteogenic differentiation of control isolated BM-MSCs. Accordingly, the P1/BM-MSCs isolated from A366-treated rats produced fewer osteocyte-like cells compared to the control (Figure 4A(Fig. 4)). Interestingly, the percentage of osteocytes was almost similar in P2/BM-MSC isolated from A366-treated rats and controls (Figure 4A(Fig. 4)). Quantitative analysis of mineralization confirmed the microscopic observation of Alizarin Red stained osteocytes. Colorimetric quantification showed that the amount of mineralized extracellular matrix was significantly lower in osteocytes differentiated from P1/BM-MSCs that were isolated from A366-treated rats compared to the control (Figure 4B, left(Fig. 4)). Although, there was approximately no difference in the amount of mineralized matrix of osteocytes differentiated from P2/BM-MSCs of both groups (Figure 4B, left(Fig. 4)). Consistence with Alizarin Red staining results, ALP activity was significantly lower in osteocytes differentiated from P1/BM-MSCs isolated from A366-treated rats (Figure 4B, right(Fig. 4)). The level of ALP activity in osteocytes derived from A366-treated rats P2/BM-MSCs, increased to the control level. We also analyzed the expression of osteogenesis specific genes in osteocytes generated from P1/BM-MSCs of both groups (Figure 4C(Fig. 4)). The relative expression of osteogenesis specific genes including Runt-related transcription factor 2 (Runx2), Osteocalcin (OCN), alkaline phosphatase (ALP), zinc finger protein Osterix (OSX) and Collagen Type I Alpha 1 Chain (Col1a1) was significantly higher in osteocytes derived from control BM-MSCs (Figure 4C(Fig. 4)).

## Discussion

Here, we used A366 to inhibit histone lysine methyltransferase G9a (EHMT2), which is an epigenetic regulator that modify key lysine and arginine residues on histones, thereby plays an important role in cancer development and maintenance. The effects of *in vivo* administrated G9a inhibitors on BM-MSC biological behaviors have not been investigated. It is known that anticancer chemicals might strongly affect other normal cells, especially MSCs that play important roles in many tissues. Therefore, we used A366 both *in vitro* (unpublished data) and *in vivo*, and analyzed the biological behaviors of BM-MSCs. It has been reported that A366 has significantly less cytotoxic effects compared to other known G9a/GLP inhibitors such as BIX01294, despite equivalent cellular activity on methylation of H3K9me2 (Pappano et al., 2015[14]). We found that *in vivo* administration of A366 heavily affected the proliferation and differentiation of BM-MSCs. We found that A366 decreased the colony-forming capacity of MSCs in primary culture, while colony-forming capacity of A366-treated and control were similar at the next passages, suggesting that the antiproliferative effect of A366 cannot sustain during *in vitro* expansion of BM-MSCs. However, it is not clear the mechanism thereby A366 decreased the number or capacity of colony-forming MSCs. Similar results were obtained from PDT assay. Accordingly, *in vivo* administration of A366 significantly prolonged PDT in the first passage, whereas PDT for BM-MSCs isolated from A366-treated rats and controls was approximately similar in the next passages, suggesting A366 effect was erased during *in vitro* expansion. The anti-proliferation effects of G9a inhibition have been reported for other cell types such as Leukemia cell lines (Pappano et al., 2015[14]). We also found that *in vivo* administration of A366 had a noticeable effect on differentiation potential of BM-MSCs. A366 increased and decreased the adipogenic and osteogenic potential of BM-MSCs, respectively. However, these effects were observed only in the first passages, suggesting that the effects of A366 cannot sustain during *in vitro* expansion. The findings of the present study are supported by previous reports indicated that H3K9me2 and G9a levels decrease during adipogenesis, which correlates inversely with induction of PPARγ (Wang et al., 2013[17]). It has been shown that G9a inhibition enhances PPARγ and C/EBPα expression and adipogenesis in preadipocytes, whereas forced expression of G9a impairs the accumulation of triglycerides (Li et al., 2013[10]). It seems that *in vivo* inhibition of G9a using A366 or other chemicals might also increase the weight of adipose tissue, and enhance the propensity of tissue specific MSCs to differentiate into adipocytes. 

We also found that G9a inhibition decreased the propensity of BM-MSCs to differentiate to osteocyte, suggesting that G9a inhibition might disturb the establishment of the gene regulatory network required for osteogenesis initiation. It has been reported that G9a interacts with Runx2 and promotes the transcriptional activation of Runx24 (Purcell et al., 2012[15]). In consistent with our findings, it has been reported that the expression of G9a is increased in osteoblasts and during prehypertrophic and hypertrophic chondrocyte formation at E16.5 (Ideno et al., 2013[8]). Therefore, inhibition of G9a using A366 or other chemicals might impair MSC involvement in tissue repair such as bone formation and remodeling. 

Interestingly, G9a inhibition has different effects on other differentiation potentials of mesenchymal cells/MSCs *in vivo* and *in vitro*. Like the inhibitory role of G9a in osteogenesis, it also works as negative regulator for myogenic differentiation. Yang and colleagues showed that inhibition of G9a by gene specific knockdown or BIX01294 treatment induced the expression of cardiogenesis specific markers such as Mesp1 and brachyury in bone marrow cells (Mezentseva et al., 2012[12]; Yang et al., 2015[18]). Moreover, G9a works as positive regulator during neurogenesis from MSCs. Kim and colleagues showed that G9a inhibitor BIX01294 increased expression of various neuronal-lineage genes in human MSCs (Kim et al., 2016[9]).

Taken together, inhibition of G9a using A366 or other chemicals might impair MSC involvement in tissue repair such as bone formation and remodeling. Although, G9a inhibition using A366 or other chemicals and approaches seems to be a strategy to control cancers, it might have unexpected and catastrophic effects on behaviors of MSCs found in several organs and tissues in the body. It will also be interesting to analyze the effects of *in vivo* administration of A366 on biological behaviors of other stem cells, as well as on MSC potential for differentiation into other lineages such as myocyte or neural cells.

## Notes

Esmail Fattahi and Mahmood Talkhabi (Department of Animal Sciences and Biotechnology, Faculty of Life Sciences and Biotechnology, Shahid Beheshti University, Tehran, Iran; Email: m_talkhabi@sbu.ac.ir) equally contributed as corrsponding authors.

## Conflict of interest

All authors declare that they have no conflict of interest. 

## Figures and Tables

**Table 1:**
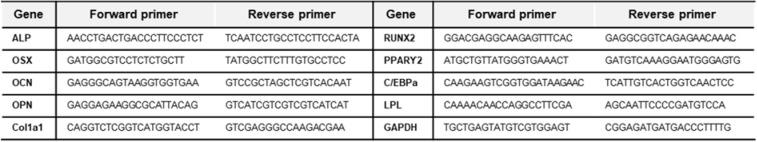
The list of primers used for real-time PCR

## References

[1] Baghaban Eslaminejad M, Nazarian H, Taghiyar L (2008). Mesenchymal stem cell isolation from the removed medium of rat’s bone marrow primary culture and their differentiation into skeletal cell lineages. Yakhteh Med J.

[2] Baghaban Eslaminejad M, Talkhabi M, Zeynali B (2008). Effect of Lithium chloride on proliferation and bone differentiation of rat marrow-derived mesenchymal stem cells in culture. Iran J Basic Med Sci.

[3] Beerman I, Rossi DJ (2015). Epigenetic control of stem cell potential during homeostasis, aging, and disease. Cell Stem cell.

[4] Casciello F, Windloch K, Gannon F, Lee JS (2015). Functional role of G9a histone methyltransferase in cancer. Front Immunol.

[5] Cho Y, Swarnabala S, Lockey RF, Kolliputi N (2015). EZH2, the moderator in the discussion between methyltransferases at histone H3?. J Cell Commun Signal.

[6] Fitzsimmons RE, Mazurek MS, Soos A, Simmons CA (2018). Mesenchymal stromal/stem cells in regenerative medicine and tissue engineering. Stem Cells Int.

[7] Ghasemzadeh N, Pourrajab F, Firoozabadi AD, Hekmatimoghaddam S, Haghiralsadat F (2018). Ectopic microRNAs used to preserve human mesenchymal stem cell potency and epigenetics. EXCLI J.

[8] Ideno H, Shimada A, Imaizumi K, Kimura H, Abe M, Nakashima K (2013). Predominant expression of H3K9 methyltransferases in prehypertrophic and hypertrophic chondrocytes during mouse growth plate cartilage development. Gene Expression Patterns.

[9] Kim H-T, Jeong S-G, Cho G-W (2016). G9a inhibition promotes neuronal differentiation of human bone marrow mesenchymal stem cells through the transcriptional induction of RE-1 containing neuronal specific genes. Neurochem Int.

[10] Li S-F, Guo L, Qian S-W, Liu Y, Zhang Y-Y, Zhang Z-C (2013). G9a is transactivated by C/EBPβ to facilitate mitotic clonal expansion during 3T3-L1 preadipocyte differentiation. Am J Physiol Endocrinol Metab.

[11] Liubaviciute A, Kaseta V, Vaitkuviene A, Mackiewicz Z, Biziuleviciene G (2018). Regenerative potential of partially differentiated mesenchymal stromal cells in a mouse model of a full-thickness skin wound. EXCLI J.

[12] Mezentseva NV, Yang J, Kaur K, Iaffaldano G, Rémond MC, Eisenberg CA (2012). The histone methyltransferase inhibitor BIX01294 enhances the cardiac potential of bone marrow cells. Stem Cells Dev.

[13] Moradi S, Sharifi-Zarchi A, Ahmadi A, Mollamohammadi S, Stubenvoll A, Günther S (2017). Small RNA sequencing reveals Dlk1-Dio3 locus-embedded microRNAs as major drivers of ground-state pluripotency. Stem Cell Rep.

[14] Pappano WN, Guo J, He Y, Ferguson D, Jagadeeswaran S, Osterling DJ (2015). The histone methyltransferase inhibitor A-366 uncovers a role for G9a/GLP in the epigenetics of leukemia. PloS One.

[15] Purcell DJ, Khalid O, Ou CY, Little GH, Frenkel B, Baniwal SK (2012). Recruitment of coregulator G9a by Runx2 for selective enhancement or suppression of transcription. J Cell Biochem.

[16] Talkhabi M, Pahlavan S, Aghdami N, Baharvand H (2015). Ascorbic acid promotes the direct conversion of mouse fibroblasts into beating cardiomyocytes. Biochem Biophys Res Commun.

[17] Wang L, Xu S, Lee JE, Baldridge A, Grullon S, Peng W (2013). Histone H3K9 methyltransferase G9a represses PPARγ expression and adipogenesis. EMBO J.

[18] Yang J, Kaur K, Ong LL, Eisenberg CA, Eisenberg LM (2015). Inhibition of G9a histone methyltransferase converts bone marrow mesenchymal stem cells to cardiac competent progenitors. Stem Cells Int.

